# Supplementary figures and images for: RNAlign2D: a rapid method for combined RNA structure and sequence-based alignment using a pseudo-amino acid substitution matrix
Source: BMC Bioinformatics. 2021 Oct 16;22:504. doi: 10.1186/s12859-021-04426-8 (PMC8520625; doi:10.1186/s12859-021-04426-8)

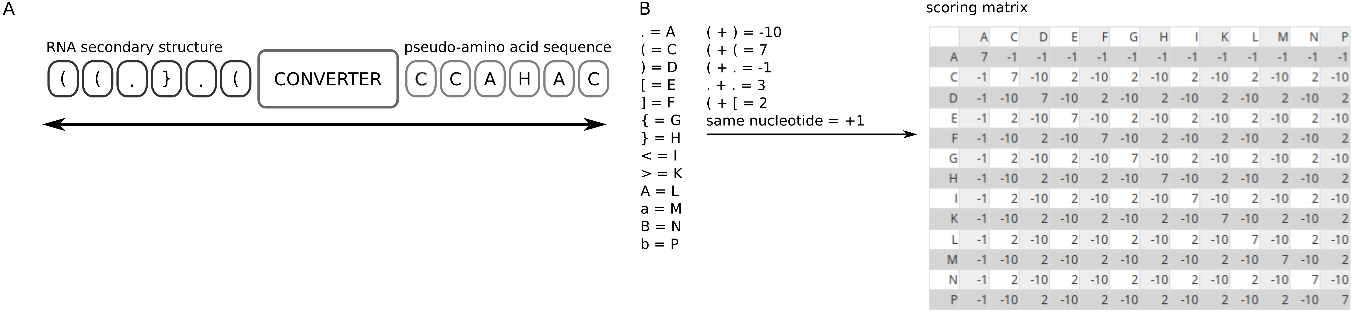

Supplement: Supplementary file 1 — Additional file 1. Figure S1. (A) Structure conversion to a pseudo-amino acid sequence for RNA with higher-level pseudoknots. (B) Conversion of structure elements to pseudo-amino acids and their scores (left) and the default scoring matrix (right). [file 12859_2021_4426_MOESM1_ESM.docx]
